# Selective Inhibition of P2Y_1_ and P2Y_12_ Receptor Signal Pathways in Platelet Aggregation in Transgenic Cell Lines and Rats by Potassium 2‐(1‐Hydroxypentyl)‐Benzoate, Puerarin and Salvianolic Acid B

**DOI:** 10.1111/cns.70089

**Published:** 2024-11-19

**Authors:** Yiying Li, Weiping Wang, Jie Cai, Nan Feng, Shaofeng Xu, Ling Wang, Xiaoliang Wang

**Affiliations:** ^1^ State Key Laboratory of Bioactive Substances and Function of Natural Medicines, Institute of Materia Medica Chinese Academy of Medical Sciences and Peking Union Medical College Beijing China; ^2^ School of Basic Medicine and Life Sciences Hainan Medical University Haikou China

**Keywords:** P2Y_1_, P2Y_12_, platelets aggregation, potassium 2‐(1‐hydroxypentyl)‐benzoate, puerarin, salvianolic acid B

## Abstract

**Aim:**

Potassium 2‐(1‐hydroxypentyl)‐benzoate (*dl*‐PHPB), puerarin and salvianolic acid B are three natural products or derivatives that can inhibit platelet aggregation. However, the mechanisms of *dl*‐PHPB, puerarin and salvianolic acid B to inhibit platelet aggregation are still not clear.

**Method:**

Here, 2‐methylthioadenosine diphosphate (2‐MeSADP) was used as an inducer to confirm the effects of three drugs on platelet aggregation and illustrate the corresponding mechanisms.

**Result:**

The results indicated that *dl*‐PHPB, puerarin and salvianolic acid B significantly inhibited platelet aggregation both in vivo and in vitro. In addition, the content of IP_3_, cAMP and intracellular [Ca^2+^]_i_ were measured in HEK293 cell lines overexpressing P2Y_1_ and P2Y_12_. *Dl*‐PHPB and puerarin could obviously reduce 2‐MeSADP‐induced IP_3_ increase, but salvianolic acid B showed no effects. Unlike *dl*‐PHPB and puerarin, which had no effects on 2‐MeSADP‐induced cAMP decrease, salvianolic acid B significantly reversed the reduction of cAMP. Both *dl*‐PHPB and puerarin could decrease the enhanced intracellular [Ca^2+^]_i_ induced by 2‐MeSADP; however, salvianolic acid B showed no effect on intracellular [Ca^2+^]_i_ elevation.

**Conclusion:**

These results suggested that *dl*‐PHPB and puerarin inhibited platelet aggregation via targeting at P2Y_1_ receptor and P2Y_1_‐Gq‐IP_3_‐Ca^2+^ signal pathway. Differently, salvianolic acid B inhibited platelet aggregation via targeting at P2Y_12_ receptor and via Gi‐AC‐cAMP signal pathway.

## Graphical abstract

*Dl*‐PHPB and puerarin inhibit the Gq‐PLC‐IP_3_‐Ca^2+^ signal transduction pathway, reduce IP_3_ and intracellular [Ca^2+^]_i_ and decrease platelet aggregation via acting on P2Y_1_ receptor. However, salvianolic acid B, like ticagrelor, inhibits the Gi‐AC‐cAMP signal transduction pathway, enhances cAMP and decreases platelet aggregation via acting on P2Y_12_.

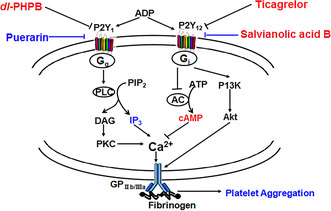

**Abbreviations**
2‐MeSADP: 2‐methylthioadenosine diphosphate
AC: adenylate cyclase
cAMP: cyclic Adenosine Monophosphate
Clo: clopidogrel
*dl*‐PHPB: patassium 2‐(1‐hydroxypentyl)‐benzoate
HEK293: human embryonic kidney cell line 293
IP_3_
: inositol 1,4,5‐triphosphate
MRS2179: *N* ^6^‐methyl‐2‐deoxyadenosine‐3, 5‐bisphosphate
PGE_1_
: prostaglandin E_1_
Tic: ticagrelor

## 1 Introduction

It is well known that platelets activation, aggregation‐induced thrombus formation is an important mechanism of acute ischemic stroke [1, 2] and it might also induce the chronic phase after acute stroke [3]. Inhibition of platelet aggregation is believed to be one of the most important strategic management in secondary prevention after a stroke [4, 5].

Some traditional Chinese herbal medicines that improve blood circulation are often used in clinics for the treatment of ischemic heart and cerebral diseases in China. 3‐n‐butylphthalide (*dl*‐NBP), puerarin and salvianolic acid B are representatives of them. They were all demonstrated to inhibit platelet aggregation and thrombus formation.

*Dl‐*NBP is purified from the seeds of *Apium graveolens* Linn and has been widely used in clinic for the treatment of ischemic stroke in China since 2005 [6, 7]. Potassium 2‐(1‐hydroxypenty1)‐benzoate (*dl*‐PHPB), undergoing a phase II clinical trial in China, is a prodrug of 3‐n‐butylphthalide. Our previous research showed that *dl*‐PHPB can effectively reduce cerebral infarct size, neuronal apoptosis, brain edema volume, stress injury, and inhibit inflammatory response [8, 9]. In addition, *dl*‐PHPB can resist platelet aggregation and thrombus formation in rats, and its mechanism is mainly through affecting the adenosine diphosphate (ADP) receptor of the platelet membrane surface [10]. The role of *dl*‐PHPB in inhibiting ADP‐induced platelet aggregation is mainly related to the P2Y_1_‐Gq‐PLC pathway [11]. However, the detailed mechanisms of *dl*‐PHPB acted on P2Y_1_ or P2Y_12_ remain unclear.

Puerarin and salvianolic acid B are the major active components of *Salvia miltiorrhiza* and Kudzu root, respectively [12, 13]. Previous studies have shown that both of them have significant therapeutic effects against ischemic stroke. Puerarin protects against ischemic brain injury via attenuating autophagy in neurons but not in astrocytes [14]. Salvianolic acid B has been proven to have anti‐inflammatory and neuroprotective effects against ischemic stroke insults in vitro and in vivo, which is associated with the inhibition of TLR4 signaling [15] and the NF‐κB pathway associated with the suppression of platelet activation and neuroinflammation [16]. However, the mechanisms of puerarin and salvianolic acid B to reduce platelet aggregation are not fully elucidated [17, 18].

Adenosine diphosphate is a substance that causes a series of physiological changes in platelets by activating ADP (purine) receptors [19]. There are two main subtypes of ADP receptor on the platelet membrane: P2Y_1_ and P2Y_12_ subtypes [20]. Currently, the ADP receptor blockers used in clinical are targeting at P2Y_12_ subtype, such as clopidogrel, ticagrelor, etc. [21]. The effect of this type of drug treatment is clear and has been used in combination with aspirin as a therapeutic and preventive drug [22]. To date, there is no anti‐platelet agent that targets the P2Y_1_ receptor has been developed.

In this study, to clarify the concise binding sites of *dl*‐PHPB, puerarin and salvianolic acid B to P2Y_1_ or P2Y_12_, we construct HEK293 cell lines overexpressing P2Y_1_ or P2Y_12_ by plasmid transient transcription. The receptor binding targets and the receptor‐signaling pathways of *dl*‐PHPB, puerarin and salvianolic acid B against platelet aggregation were then compared. Clarifying the mechanisms of the compounds might be helpful in developing new class anti‐platelet drugs from natural products.

## 2 Materials and Methods

### 2.1 Animals

Male Sprague Dawley (SD) rats (weight 220–240 g) were housed in a room with a 12‐h light/dark cycle, fed with normal rat chow and water ad libitum. All experiments were performed in accordance with institutional guidelines of the Experimental Animal Center of the Chinese Academy of Medical Science.

### 2.2 Drugs and Reagents

*Dl*‐PHPB was provided by the Department of Synthetic Pharmaceutical Chemistry, Institute of Materia Medica, Chinese Academy of Medical Sciences, with a purity of 99.9%. *dl*‐PHPB was dissolved in deionized water. Puerarin was purchased from Beijing Union Pharmaceutical Factory and was dissolved in 0.5% CMC‐Na solution for oral administration and DMSO for incubation. Salvianolic acid B was purchased from Tasly Phar. (Tianjing, China) and was dissolved in deionized water. The human embryonic kidney (HEK 293) cell line was preserved in our laboratory. pCDNA3.1‐flag, pCDNA3.1‐flag tagged human P2Y_1_ receptor and pCDNA3.1‐flag tagged human P2Y_12_ receptor were purchased from Biogot technology, Co. Ltd. Rat P2Y_1_ antibody, rat P2Y_12_ antibody, 2‐MeSADP, ADP and *N* ^6^‐methyl‐2‐deoxyadenosine‐3, 5‐bisphosphate (MRS2179) were purchased from Sigma Chemical Co. (St. Louis, MO, USA). Inositol 1,4,5‐triphosphate kit (IP_3_ kit) was purchased from Wuhan BOSTER. Ltd. Cyclic. Adenosine Monophosphate kit (cAMP kit) was purchased from Wuhan USCN Business Co. Ltd., Ticagrelor (Tic) was purchased from Toronto Research Chemicals (North York, Canada). Clopidogrel (Clo) was purchased from the National Institutes for Food and Drug Control.

### 2.3 Transient Transfection of pcDNA3.1‐P2Y_1_ and pcDNA3.1‐P2Y_12_ Eukaryotic Expression Vectors Into HEK 293 Cells

HEK293 cells were transduced with various combinations of plasmid using the Lipofectamine LTX and Plus reagents (Invitrogen, CA) according to the manufacturer's protocol. Briefly, cells were seeded 24‐h prior to transfection into a 6‐well plate at a density of 1 × 10^5^/well. One 1 μL of DNA was mixed with 2.5 μL of Lipofectamine LTX and 1 μL of Plus reagent in 200 μL of OPTI‐MEM (Invitrogen) medium, incubated at room temperature for 30 min, and then added to each well of the 6‐well plate.

### 2.4 Cell Culture

The cell line used in this study was maintained in Dulbecco's modified Eagle medium (DMEM, Invitrogen) supplemented with 10% fetal bovine serum (FBS, Gibco), 1% penicillin/streptomycin (Invitrogen) at 37°C in a humidified 5% CO_2_ environment.

### 2.5 Western Blotting

Protein expression in HEK 293 cells was determined by Western blotting. Briefly, after HEK 293 cells were transfected for 48 h, they were pipetted with medium and washed once with 0.01 M PBS. Followed by lysing the cells into ice‐cold lysis buffer (150 mM NaCl, 10 mM Tris–HCl (pH 7.0), 1 mM EGTA, 1% (v/v) NP40, 10% (v/v) glycerol, 1 mg/mL cocktail). Samples were centrifuged at 12,000 rpm for 30 min at 4°C. Protein concentrations were determined by the Bradford method.

Protein samples were separated by 10% SDS/PAGE gels and transferred to a PVDF membrane. Then, blocking the membranes at room temperature in 5% BSA with TBST buffer (10 mM Tris–HCl, 120 mM NaCl, 0.1% Tween‐20, pH 7.4) for 2 h or more, followed by incubating with primary antibody (1:1000 in TBST) at 4°C overnight. The membranes were then washed in TBST buffer (five times/5 min one time), followed by incubation with HRP‐conjugated secondary antibody at room temperature for 2 h, and washed by TBST five times. The density of the bands on western blots was quantified using Bio‐rad Quantity‐One software. The values were normalized to β‐actin intensity levels.

### 2.6 Immunofluorescence Identification

After 48 h, transfected HEK 293 cells were washed twice with 0.01 M PBS, and then fixed by 4% PFA at room temperature for 1 h, followed by washing with PBS 3times. 5% goat serum (using TBS, TBS: PBS + 0.5% Triton) was blocked at room temperature for 30 min; then add mouse anti‐Flag (1:200 dilution) antibody, and incubated overnight at 4°C. Finally, the sheep anti‐mouse FITC‐IgG (1:500 dilutions) was added, and then incubated for 2 h at room temperature; Observe and collect the images under a fluorescence microscope. The excitation/emission wavelength of FITC is green fluorescence at 488/525 nm.

### 2.7 Platelet Aggregation

For in vivo measurement, rats were anesthetized with 10% chloral hydrate (3 mL/kg, i.p.) and blood was collected from the abdominal aorta and anti‐coagulated with sodium citrate (3.8%; 1:9, v/v). Platelet‐rich plasma (PRP) was obtained by centrifuging blood at 1000 rpm for 10 min at room temperature. The remainder was further centrifuged at 3000 rpm for 10 min to obtain platelet‐poor plasma (PPP). Washed platelets were prepared as previously described [23]. The platelet counts of PRP were adjusted to 4 × 10^8^/mL with PPP. For in vitro measurements, blood was collected from normal SD rats, and rat platelets were pre‐incubated with tested drugs. Platelet aggregation was determined using a four‐channel aggregometer (LBY‐NJ4, Beijing Precil Instrument Co. Ltd., China). 300 μL PRP was placed in a cuvette and stirred with a rotor at 37°C for 5 min, 2‐MesADP (0.1 μM) was then added. Platelet aggregation was determined by calibrating the equipment at 0% light transmission for PRP and at 100% for PPP. All experiments were conducted within 2 h of blood collection. The aggregation curve was recorded for 5 min and the peak value was received and served as the maximal aggregation (MAG). Inhibition% of platelet aggregation was calculated from the formula:

$$\begin{array}{cc} \text{Inhibition}\%= & \left(\mathrm{MAG}\;\text{of control}-\mathrm{MAG}\;\text{of tests}\right)/\mathrm{MAG}\;\text{of control}\times \\ & 100\% \end{array}$$

### 2.8 Measurement of IP_3_ Concentration

The cells were transfected for 48 h and then added with tested drugs. After being labeled for 15 min, the cells were stimulated with 2‐MeSADP (final concentration 0.1 μM). Five minutes later, the reaction was stopped in an ice bath and added cell lysate. Samples were centrifuged at 12,000 rpm for 30 min at 4°C. IP_3_ were determined by enzyme immunoassay kit according to the manufacturer's procedure (CUSABIO, Wuhan, China).

### 2.9 Measurement of cAMP Concentration

The cells were transfected for 48 h and then added with tested drugs. After incubation, the cells were stimulated with 2‐MeSADP (final concentration of 0.1 μM). After 5 min, the cells were washing twice with PBS, lysed with 0.1 N HCl, scraped, and collected by centrifugation. The content of cAMP in the supernatants was determined using a cyclic AMP EIA kit according to the manufacturer's instructions.

### 2.10 Measurement of Intracellular Ca^2+^ Concentration

Forty‐eight hours after transfection, the medium was discarded. The suspended transfected cells were loaded with 2 μM Fluo‐4‐acetoxymethyl ester (Invitrogen) in 100 μL of Hanks' balanced salt solution buffered with 20 mM HEPES, pH 7.5 (HBSS), the cells were incubated at 37°C for 1 h in the dark. After centrifuging the remains were washed twice with HBSS‐HEPES solution and resuspended in HBSS‐HEPES at a cell density of 4 × 10^8^/mL. The cells were incubated with an antagonist for 30 min before treatment with 2‐MeSADP (final concentration of 0.1 μM). All manipulations were performed with CytoFLEX flow cytometry (Beckman) at Ex at 488/Em at 525 nm to measure intracellular Ca^2+^.

### 2.11 Molecular Docking

The X‐ray structures of P2Y1 (PDB: 4xnw) and P2Y12 (PDB: 4PXZ) were downloaded from the PDB. Firstly, all the water molecules and irrelevant ligands were removed from the crystal structures. Then the structures were prepared with the “Clean Protein” module of Discovery Studio 2016. As a result, hydrogen atoms were added, chain termini were modified, incomplete residues were repaired and the whole protein structure was protonated at the pH of 7.0. All the water molecules were removed from the crystal structures. GOLD (version 3.0.1, Cambridge Crystallographic Data Center, Cambridge, UK) was the program for molecular docking in this study. The settings for all the three docking simulations were the same. The binding site was defined as a sphere centered on the cognate ligand with a radius of 10 Å. The times for molecular docking were set to 20. The scoring function to predict protein‐ligand affinity was GoldScore. The binding poses generated by GOLD were visually inspected and the plausible binding pose was retained.

The protein structure processing is completed by the protein preparation module in Schr ö dinger software. The downloaded P2Y_1_ and P2Y_12_ target crystal structures (4xnw, 4PXZ) were introduced into Schrodinger, respectively, and the chemical bond in the protein structure was modified. Then, handling of metal ions, hydrogenation, neutralization of the amino acid residues at the N and C ends was proceeded, retaining the original ligands and proteins to remove impurity atoms and water molecules. We optimized the protein structure by selecting OPLS_2005 and chose sample water orientations as the parameter to optimize the hydrogen bond, and the convergence standard was RMSD 0.5 Å.

Apply the LigPrep module to optimize small molecules, remove salt, add charge, ionize at pH = 7 ± 2, and optimize the molecular force field to OPLS_2005, the molecular Tautomer was formed. In stereoisomerism processing, the method of generating chirality according to the input stereo structure is adopted, and others are the default parameters.

Macromolecular docking is carried out using the “docking” module in Schrodinger software. Select “receptor box generation”, and take the original ligand small molecule as the box center to automatically generate the receptor box file. Standard precision flexible docking was adopted.

### 2.12 Statistical Analysis

Data were expressed as mean ± SEM with a 95% confidence interval. Except for the WB experiment, which was only repeated 3 times, all other experiments were repeated 4–5 times. Statistical analysis was performed with SPSS 13.0 software (SPSS Inc. Chicago, IL, USA). The normality distribution of all data was tested with a Shapiro‐Wilk test. Student's *t*‐test was used to compare the two groups. Multiple samples with normal distribution and homogeneous variance were compared with a one‐way analysis of variance (ANOVA) with an LSD test. *p* < 0.05 was considered to be statistically significant.

## 3 Results

### 3.1 Effects of *Dl*‐PHPB, Puerarin and Salvianolic Acid B on 2MeSADP‐Induced Rat Platelet Aggregation In Vivo and In Vitro

**FIGURE 1:**
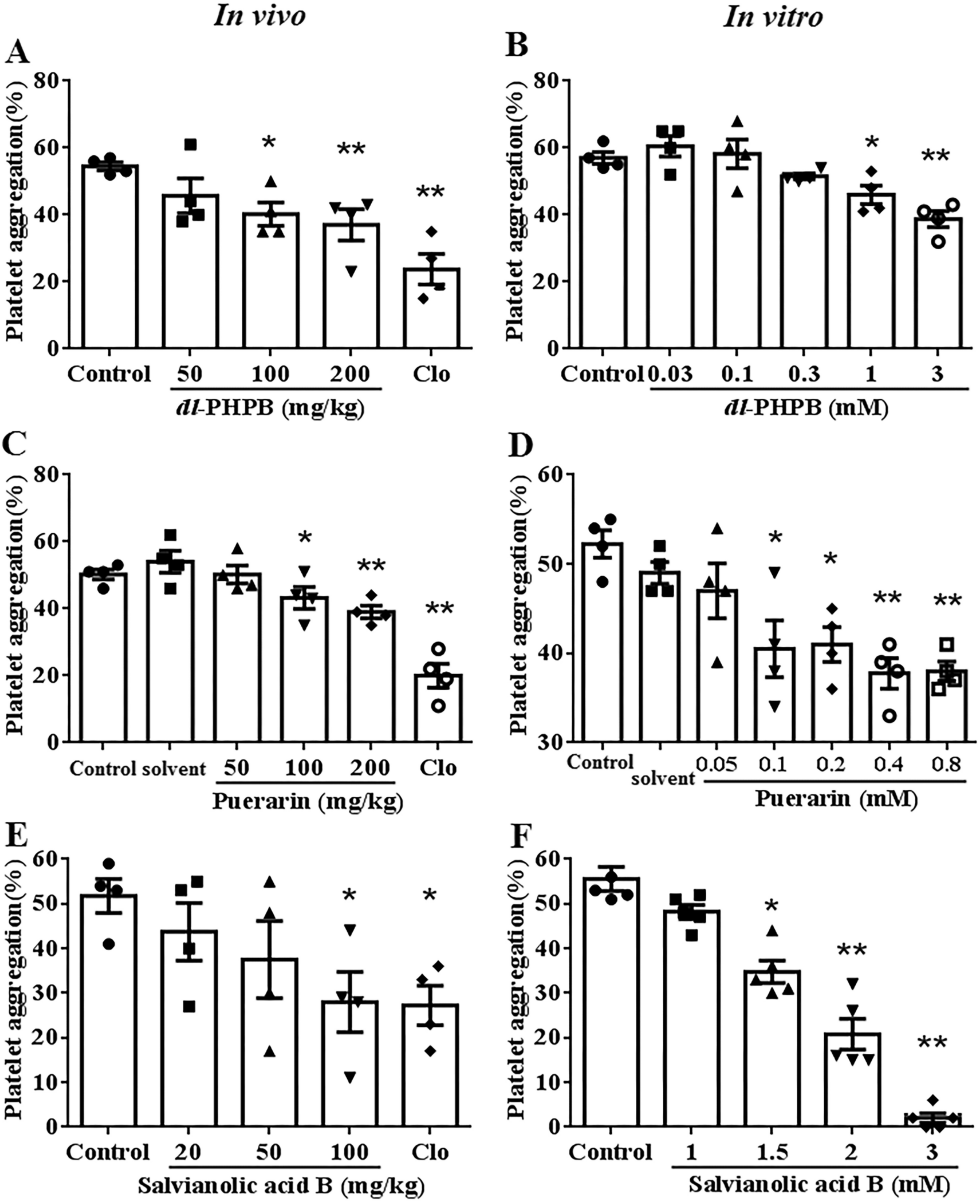
Effects of *dl*‐PHPB, puerarin and salvianolic acid B on 2MeSADP‐induced rat platelet aggregation in vivo and in vitro. For in vivo assessment, rats were treated with *dl*‐PHPB (A), puerarin (C) and salvianolic acid B (E) or clopidogrel (10 mg/kg) by oral gavage. Rat blood was collected 1 h after orally gavage. The solvents were deionized water for *dl*‐PHPB and salvianolic acid B, and 0.5% CMC‐Na for puerarin. For in vitro assessment, rat blood was collected and rat platelets were pre‐incubated with *dl*‐PHPB (B), puerarin (D) and salvianolic acid B (F) for 5 min. The solvents were ddH_2_O for *dl*‐PHPB and salvianolic acid B, and DMSO for puerarin (The final concentration of DMSO was 0.1%). Platelet aggregation was induced by 0.1 μM 2‐MeSADP. The aggregation was assessed using a four‐channel aggregometer. *n* = 4 for (A–E) and *n* = 5 for (F), *p < 0.05, **p < 0.01 versus control or solvent.

For in vivo measurements, *dl*‐PHPB, puerarin and salvianolic acid B were orally administrated to rats, and ex vivo platelet aggregation induced by 0.1 μm 2‐MeSADP was then measured 30 min after each administration. *Dl*‐PHPB, puerarin and salvianolic acid B dose‐dependently inhibited 2MeSADP‐induced platelet aggregation. *Dl*‐PHPB at 50, 100 and 200 mg/kg decreased 2‐MeSADP‐induced platelet aggregation from 54.5% ± 1.2% to 45.8% ± 5.2%, 40.3% ± 3.5% and 37% ± 4.7%, respectively (Figure 1A, data were obtained from 4 rats, *p < 0.05, **p < 0.01 vs. control). Puerarin at 50, 100 and 200 mg/kg decreased 2‐MeSADP‐induced platelet aggregation from 54% ± 3.3% to 50.3% ± 2.7%, 43.3% ± 3.3% and 39% ± 1.9, respectively (Figure 1C, data were obtained from 4 rats, *p < 0.05, **p < 0.01 vs. solvent). Salvianolic acid B at 20, 50 and 100 mg/kg decreased 2‐MeSADP‐induced platelet aggregation from 51.8% ± 3.8% to 43.8% ± 6.5%, 37.5% ± 8.6% and 28% ± 6.8%, respectively (Figure 1E, data were obtained from 4 rats, *p < 0.05 vs. control). Clopidogrel at 10 mg/kg showed more potent effects on 2‐MeSADP‐induced platelet aggregation than *Dl*‐PHPB and puerarin at 200 mg/kg and the same effect as salvianolic acid B at 100 mg/kg.

For in vitro measurements, blood was collected from normal SD rats and rat platelets were pre‐incubated with *dl*‐PHPB, puerarin and salvianolic acid B for 5 min, respectively. *Dl*‐PHPB, puerarin and salvianolic acid B significantly inhibited platelet aggregation induced by 2‐MeSADP in a dose‐dependent manner. *Dl*‐PHPB at 0.03, 0.1, 0.3, 1 and 3 mM decreased 2MeSADP‐induced platelet aggregation from 57% ± 1.8% to 60.5% ± 3.1%, 58.3% ± 4.3%, 51.5% ± 0.9%, 46% ± 2.8% and 38.8% ± 2.4%, respectively (Figure 1B, *n* = 4, *p < 0.05, **p < 0.01 vs. control). Puerarin at 0.05, 0.1, 0.2, 0.4 and 0.8 mM reduced 2MeSADP‐induced platelet aggregation from 49% ± 1.2% to 47.00% ± 3.08%, 40.50% ± 3.18%, 41.00% ± 1.96%, 37.75% ± 1.70% and 38.00% ± 1.08%, respectively (Figure 1D, *n* = 4, *p < 0.05, **p < 0.01 vs. solvent). Salvianolic acid B at 1, 1.5, 2 and 3 mg/kg decreased 2MeSADP‐induced platelet aggregation from 55.6% ± 2.7% to 48.20% ± 1.59%, 34.8% ± 2.52%, 20.80% ± 3.48% and 2.00% ± 1.10%, respectively (Figure 1F, *n* = 5, *p < 0.05, **p < 0.01 vs. control).

These results suggested that *dl*‐PHPB, puerarin and salvianolic acid B could significantly inhibit platelet aggregation induced by 2‐MeSADP both in vivo and in vitro.

### 3.2 Identification of Protein Expression of P2Y_1_ and P2Y_12_ Transiently Transfected in HEK293 Cells

**FIGURE 2:**
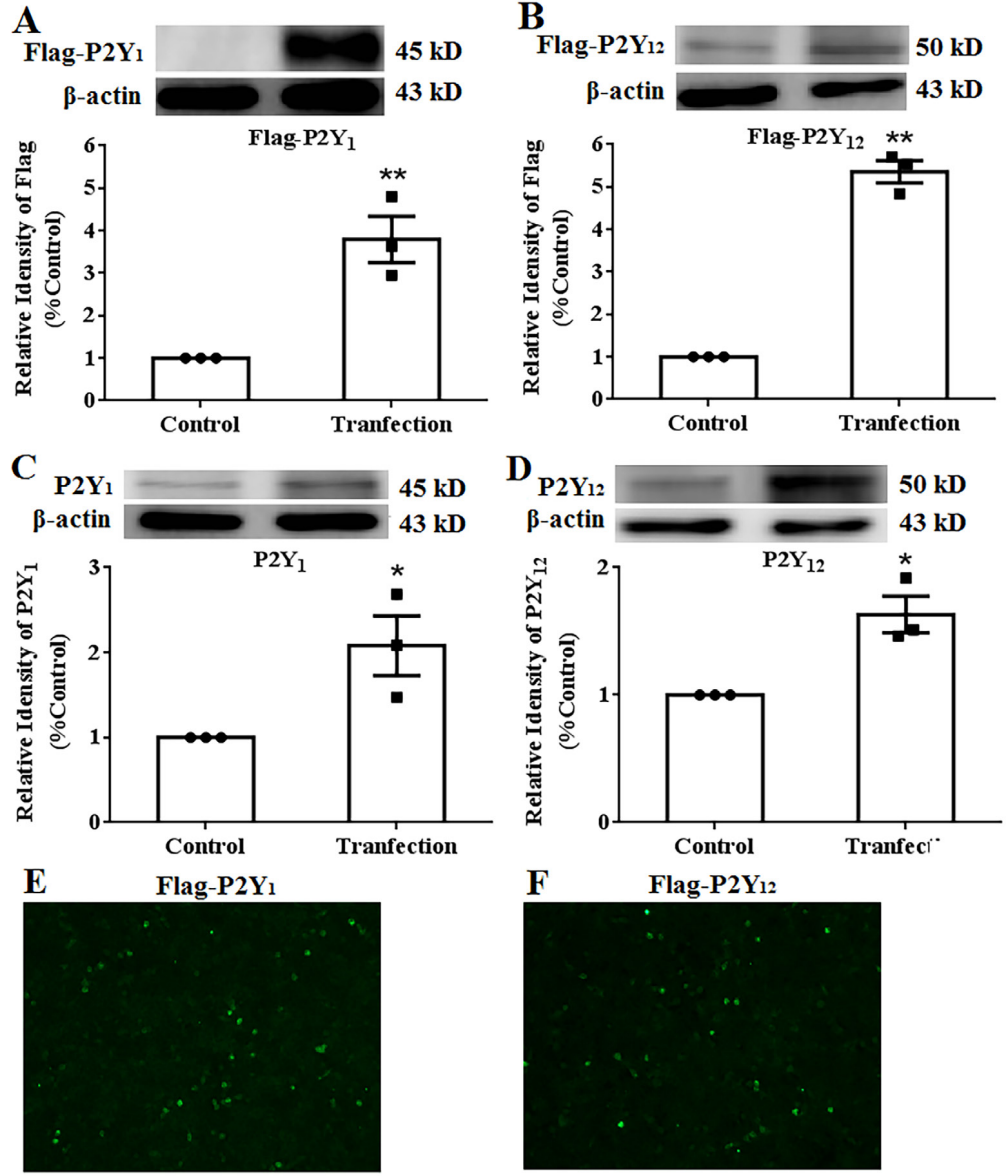
Identification of protein expression of P2Y_1_ and P2Y_12_ transiently transfected in HEK293 cells. The expression of Flag in HEK293 cells was elevated after pcDNA‐P2Y_1_‐Flag (A) and pcDNA‐P2Y_12_‐Flag (B) were transfected. The expression of P2Y_1_ (C) and P2Y_12_ (D) in HEK293 cells were elevated after pcDNA‐P2Y_1_‐Flag and pcDNA‐P2Y_12_‐Flag were transfected. Typical immunofluorescence images after P2Y_1_ (E) and P2Y_12_ (F) transfection. Quantified results were normalized to β‐Actin expression. Values were expressed as percentages compared to the control group (set to 100%). *n* = 3, *p < 0.05, ^**^ *p* < 0.01 versus control.

To illustrate the underlying mechanisms of *dl*‐PHPB, puerarin and salvianolic acid B on platelet aggregation, P2Y_1_ or P2Y_12_ were transiently transfected in HEK293 cells. Flag protein expression was obviously observed both in pcDNA3.1‐P2Y_1_ and pcDNA3.1‐P2Y_12_ transfected cells (Figure 2A,B, *n* = 3, **p < 0.01 vs. control). It indicated that HEK293 cells can express P2Y_1_ or P2Y_12_ protein efficiently after 48 h of transfection. In addition, the expression of P2Y_1_ was significantly increased both in pcDNA3.1‐P2Y_1_ and pcDNA3.1‐P2Y_12_ transfected group, which was significantly higher than that in the empty plasmid group (Figure 2C,D, *n* = 3, *p < 0.05 vs. control). It is indicated that HEK293 cells can overexpress P2Y_1_ or P2Y_12_ protein efficiently after 48 h of transfection. The immunofluorescence analysis further confirmed that HEK293 cells could express P2Y_1_ or P2Y_12_ protein with great efficiency (Figure 2E,F). These results suggested that transient transfection of P2Y_1_ and P2Y_12_ in HEK293 cells was successful.

### 3.3 Identification of IP_3_ , cAMP and [Ca^2+^]_i_ Measurements in HEK293 Cells Transfected With P2Y_1_ and P2Y_12_

**FIGURE 3:**
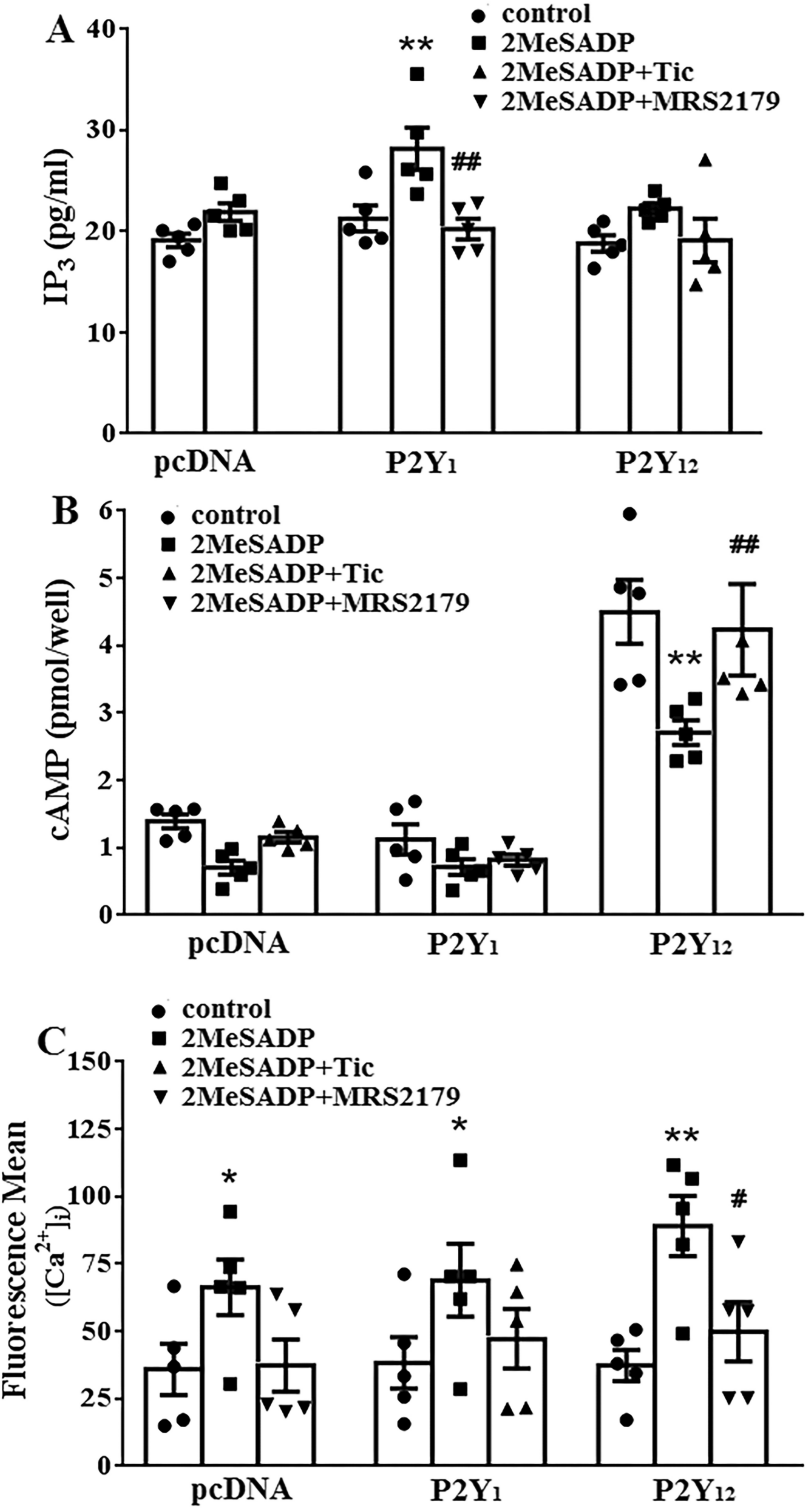
Identification of IP3, cAMP and [Ca^2+^]_i_ measurements in HEK293 cells transfected with P2Y_1_ and P2Y_12_ pcDNA3.1, pcDNA3.1‐P2Y_1_, pcDNA3.1‐P2Y_12_ was transfected into HEK293 cells to measure the content of IP_3_, cAMP and [Ca^2+^]_i_ in vitro. 0.3 μM P2Y_1_ antagonist MRS2179 or 0.5 μM P2Y_12_ antagonist ticagrelor before stimulated with 2MeSADP were used as positive drugs to verify the reliability of the measuements of IP_3_ (A), cAMP (B) and [Ca^2+^]_i_ (C). IP_3_ and cAMP were determined by immunoassay. [Ca^2+^]_i_ was determined by a flow cytometry. *n* = 5, *p < 0.05, **p < 0.01 versus control group, ^#^ *p* < 0.05, ^##^ *p* < 0.01 versus 2MeSADP.

Gq‐PLC‐IP_3_ and Gi‐AC‐cAMP signal pathways are closely related to platelet aggregation, IP_3_, cAMP and [Ca^2+^]_i_ are important downstream second massagers. Therefore, positive inhibitors of P2Y_1_ or P2Y_12_ receptors were used to verify the reliability of IP_3_, cAMP and [Ca^2+^]_i_ measurements in HEK293 cells transfected with P2Y_1_ or P2Y_12_. For IP_3_ measurement, 0.1 μM 2‐MeSADP significantly increased IP_3_ content in the HEK 293 cells transfected with P2Y_1_, while after incubating with P2Y_1_ receptor antagonist MRS2197 (0.3 μM), the IP_3_ content was clearly reduced (Figure 3A, *n* = 5, **p < 0.01 vs. control, ^##^ *p* < 0.01 vs. 2‐MeSADP). For cAMP measurement, 0.1 μM 2‐MeSADP significantly decreased cAMP content in the HEK 293 cells transfected with P2Y_12_, while after incubating with P2Y_12_ receptor antagonist ticagrelor (0.5 μM), the cAMP content was increased accordingly (Figure 3B, *n* = 5, **p < 0.01 vs. control, ^##^ *p* < 0.01 vs. 2‐MeSADP). For [Ca^2+^]_i_ measurement, 0.1 μM 2‐MeSADP significantly increased intracellular [Ca^2+^]_i_ in the HEK 293 cells transfected with P2Y_1_, while after incubating with P2Y_1_ receptor antagonist MRS2197 (0.3 μM), the increased intracellular [Ca^2+^]_i_ was clearly decreased (Figure 3C, *n* = 5, *p < 0.05, **p < 0.01 vs. control, ^#^ *p* < 0.05 vs. 2‐MeSADP). These results indicated that MRS2197 and ticagrelor can inhibit the binding of 2‐MeSADP to P2XY_1_ and P2Y_12_, respectively, which is consistent with the role of MRS2197 and ticagrelor as corresponding recognized P2XY_1_ and P2Y_12_ receptor antagonist. The IP_3_, cAMP and [Ca^2+^]_i_ measurements in HEK293 cells transfected with P2Y_1_ or P2Y_12_ are reliable.

### 3.4 Effects of *Dl*‐PHPB, Puerarin and Salvianolic Acid B on IP_3_ Content in HEK293 Cells Transfected With P2Y_1_

**FIGURE 4:**
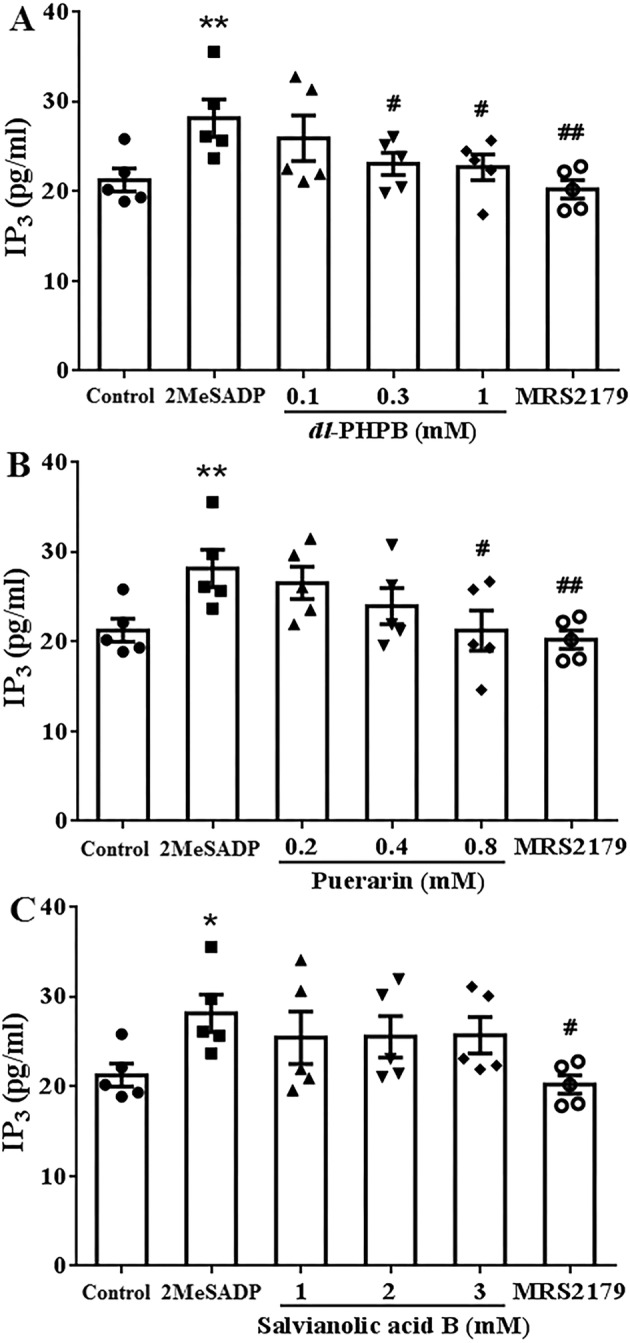
Effects of *dl*‐PHPB, puerarin and salvianolic acid B on 2‐MeSADP‐induced IP_3_ increase in HEK 293 cell transfected with P2Y_1_. The IP_3_ content was enhanced by 0.1 μM 2‐MeSADP and could be reversed by 0.5 μM MRS2179. *dl*‐PHPB (A), puerarin (B) and salvianolic acid B (C) were preincubated before stimulated with 2‐MeSADP. IP_3_ content was determined by immunoassay. *n* = 5, *p < 0.05, **p < 0.01 versus control group, ^#^ *p* < 0.05, ^##^ *p* < 0.01 versus 2MeSADP.

After adding 0.1 μM 2‐MeSADP into HEK 293 cells transfected with P2Y_1_, the IP_3_ content of the cells were significantly increased (Figure 4, *n* = 5, *p < 0.05, **p < 0.01 vs. control). Whereas there was a significant decrease in IP_3_ after incubation with 0.3 μM MRS2179, the IP_3_ content was decreased from 28.2 ± 2.1 pg/mL to 20.2 ± 1.0 pg/mL (Figure 4, *n* = 5, ^#^ *p* < 0.05, ^##^ *p* < 0.01 vs. 2‐MeSADP). *Dl*‐PHPB 0.3 mM and 1 mM reduced the IP_3_ content to 23.06 ± 1.25 pg/mL and 22.68 ± 11.42 pg/mL, respectively (Figure 4A, *n* = 5, ^#^ *p* < 0.05 vs. 2‐MeSADP). A similar result could be found after being incubated with puerarin 0.8 mM in vitro (Figure 4B, *n* = 5, ^#^ *p* < 0.05 vs. 2‐MeSADP). Unlike *dl*‐PHPB and puerarin, salvianolic acid B showed no effects on IP_3_ content (Figure 4C, *n* = 5). These results suggested that salvianolic acid B‐inhibited platelet aggregation might be not via Gq‐PLC‐IP_3_ signal pathway.

### 3.5 Effects of *Dl*‐PHPB, Puerarin and Salvianolic Acid B on cAMP Content in HEK293 Cells Transfected With P2Y_12_

**FIGURE 5:**
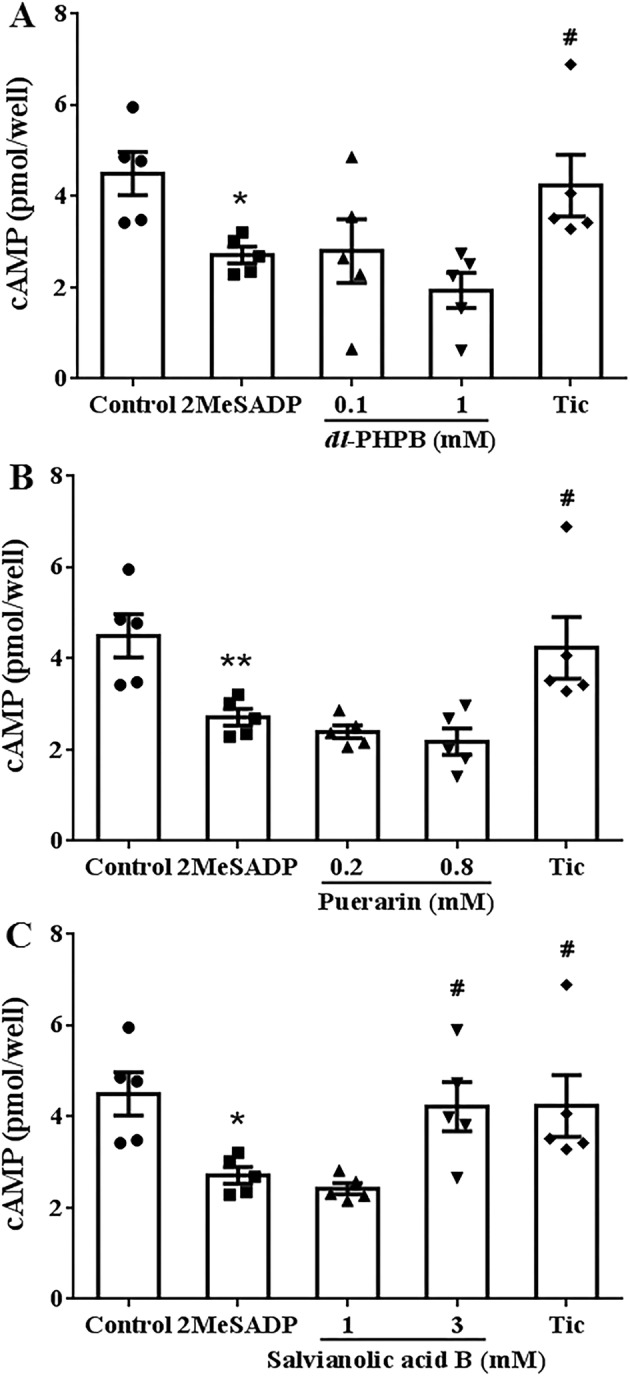
Effects of *dl*‐PHPB, puerarin and salvianolic acid B on 2MeSADP‐induced cAMP decrease in HEK 293 cell transfected with P2Y_12_. The cAMP content was reduced by 0.1 μM 2MeSADP and could be reversed by 0.5 μM ticagrelor. *dl*‐PHPB (A), puerarin (B) and salvianolic acid B (C) were preincubated before stimulated with 2‐MeSADP. cAMP content was determined by immunoassay. *n* = 5, *p < 0.05, **p < 0.01 versus control group, ^#^ *p* < 0.05 versus 2MeSADP.

The cAMP is a “second messenger” in platelets and plays a crucial role in the activation and function of platelets. Because *dl*‐PHPB and puerarin decreased the IP_3_ content, we further test the effects of *dl*‐PHPB, puerarin and salvianolic acid B on the cAMP content. After adding 0.1 μM 2‐MeSADP into HEK 293 cells transfected with P2Y_12_, the cAMP content was significantly decreased and could be reversed by 0.5 μM ticagrelor (Figure 5, *n* = 5, ^#^ *p* < 0.05, ^##^ *p* < 0.01 vs. control, *p < 0.05 vs. 2‐MeSADP). There was no significant change in cAMP content after preincubation with *dl*‐PHPB and puerarin in vitro (Figure 5A,B, *n* = 5). Unlike *dl*‐PHPB and puerarin, salvianolic acid B at 3 mM obviously reversed the reduction of cAMP induced by 2‐MesADP (Figure 5C, *n* = 5, ^#^ *p* < 0.05 vs. 2‐MeSADP). The above results showed that different from salvianolic acid B, *dl*‐PHPB and puerarin do not inhibit platelet aggregation by increasing cAMP content.

### 3.6 Effects of *Dl*‐PHPB, Puerarin and Salvianolic Acid B on Intracellular Ca^2+^ Concentration in HEK293 Cells Transfected With P2Y_12_

**FIGURE 6:**
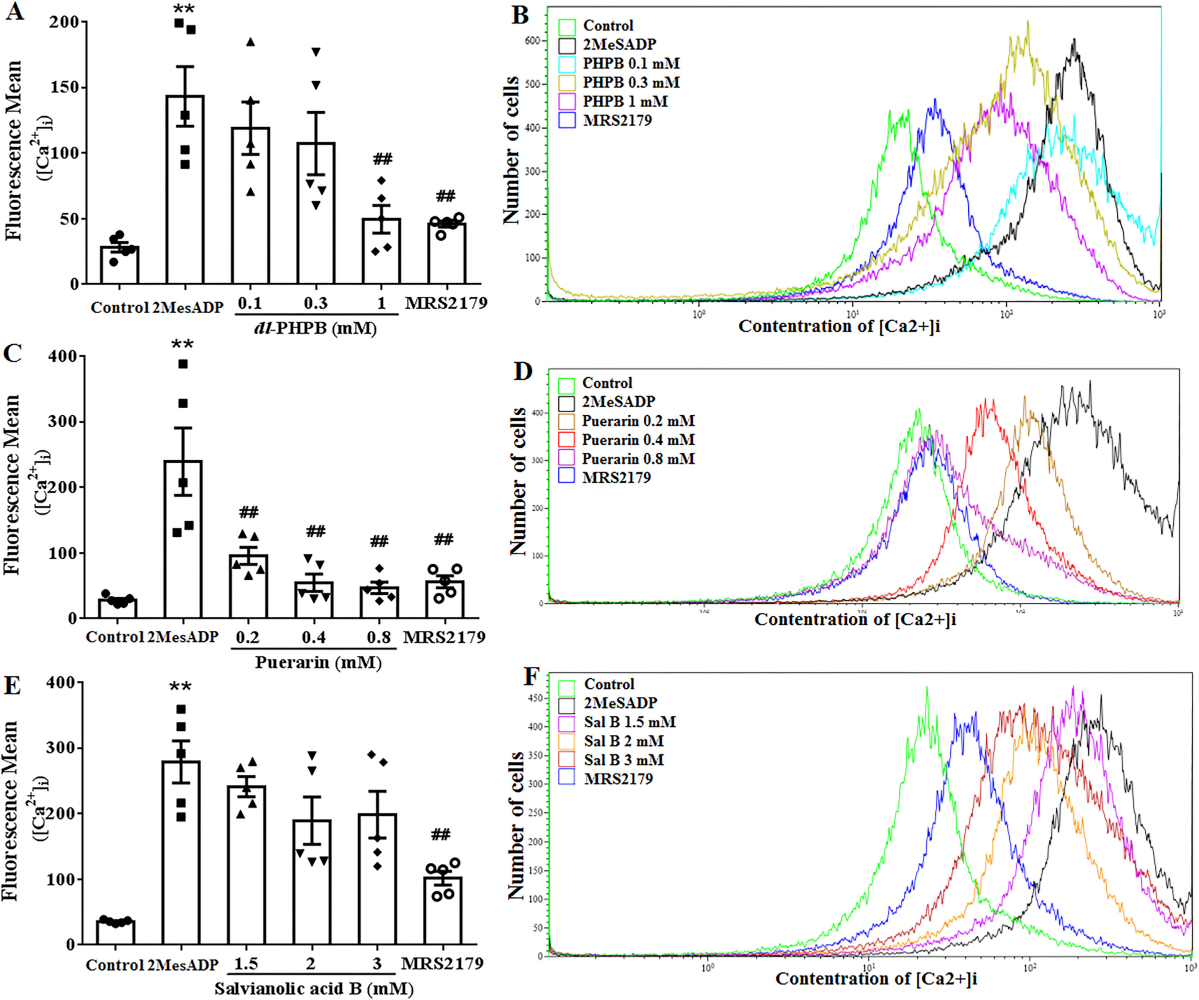
Effects of *dl*‐PHPB, puerarin and salvianolic acid B on 2MeSADP‐induced intracellular [Ca^2+^]i increase in HEK 293 cells transfected with P2Y_1_. The intracellular [Ca^2+^]i was increased by 0.1 μM 2MeSADP and could be reversed by 0.5 μM MRS2179. *Dl*‐PHPB (A), puerarin (C) and salvianolic acid B (E) were preincubated before stimulated with 2‐MeSADP. The intracellular [Ca^2+^]i was determined by a flow cytometry. Typical images of *dl*‐PHPB (B), puerarin (D) and salvianolic acid B (F) from flow cytometry were shown. *n* = 5, **p < 0.01 versus control group, ^##^ *p* < 0.01 versus 2MeSADP.

Another crucial factor in platelet aggregation is the change of Ca^2+^, thus we tested whether *dl*‐PHPB, puerarin and salvianolic acid B suppressed 2‐MeSADP‐induced Ca^2+^ mobilization or not. After adding 0.1 μM 2‐MeSADP into HEK 293 cells transfected with P2Y_12_, the intracellular [Ca^2+^]_i_ was significantly increased and could be reversed by 0.3 μM 2‐MeSADP (Figure 6, *n* = 5, **p < 0.01 vs. control, ^##^ *p* < 0.01 vs. 2‐MeSADP). Preincubation with 1 mM *dl*‐PHPB and 0.2–0.8 mM puerarin significantly inhibited 2‐MeSADP‐induced intracellular [Ca^2+^]_i_ elevation (Figure 6A,B, *n* = 5, ^##^ *p* < 0.01 vs. 2‐MeSADP). Unlike *dl*‐PHPB and puerarin, salvianolic acid B showed no effect on 2‐MeSADP‐induced intracellular [Ca^2+^]_i_ elevation (Figure 6E, *n* = 5). These results further proved that *dl*‐PHPB and puerarin inhibit platelet aggregation via Gq‐PLC‐IP_3_ pathway, which was different from salvianolic acid B via Gi‐AC‐cAMP pathway.

### 3.7 Molecular Docking of *Dl*‐PHPB, Puerarin and Salvianolic Acid B With P2Y_1_ and P2Y_12_

**FIGURE 7:**
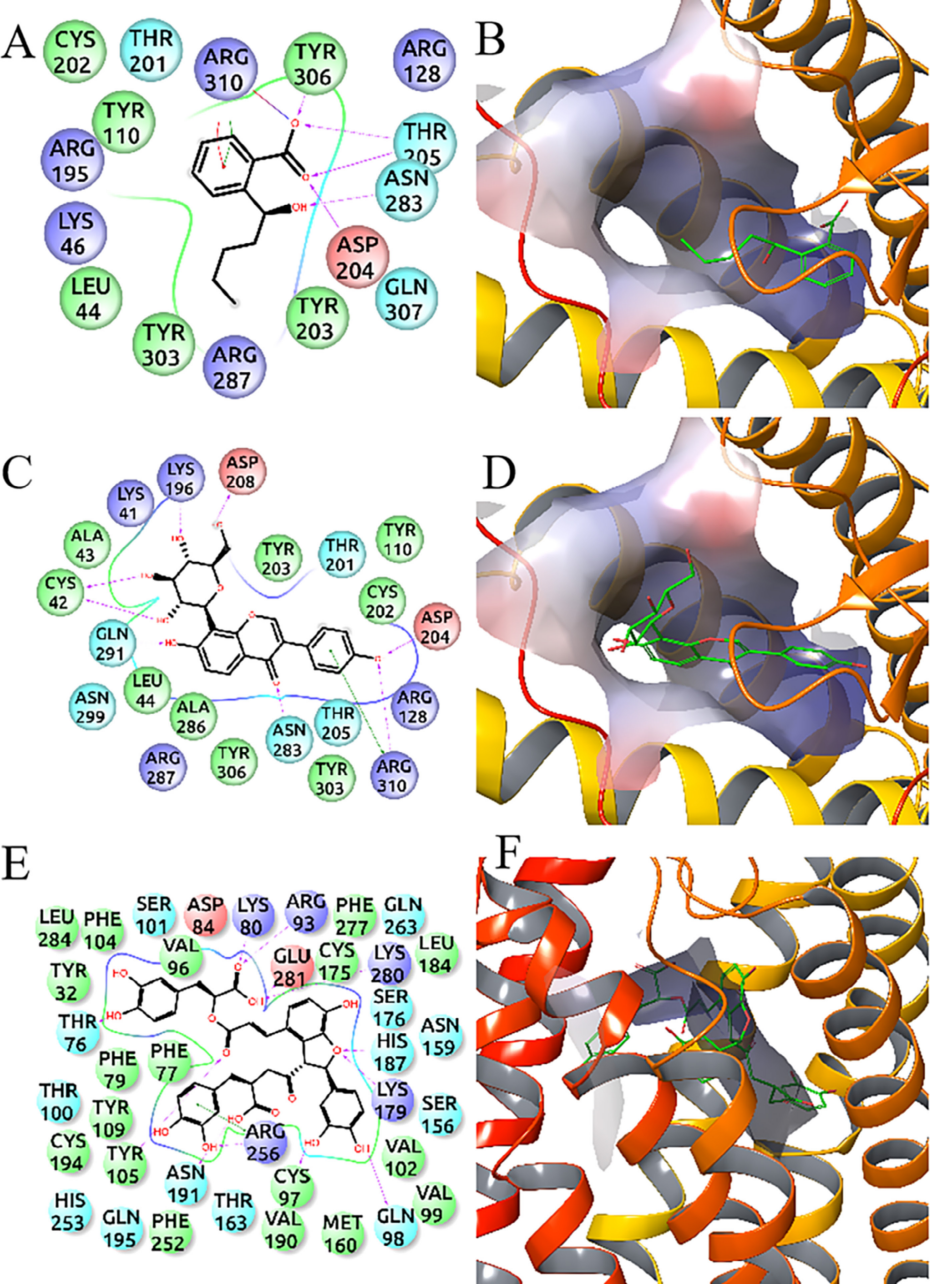
The molecular docking of *dl*‐PHPB, puerarin and salvianolic acid B with P2Y_1_ and P2Y_12_ receptor. The computer molecular docking was shown that the bond energies of *dl*‐PHPB and puerarin with P2Y_1_ receptor were − 7.250 and − 6.468 kcal/mol, while the bond energy salvianolic acid B with P2Y_12_ receptor was −4.039 kcal/mol. (A, B) Two‐dimensional interaction map and three‐dimensional docking pose of *dl*‐PHPB with P2Y_1_ receptor. (C, D) Two‐dimensional interaction map and three‐dimensional docking pose of puerarin with P2Y_1_ receptor. (E, F) Two‐dimensional interaction map and three‐dimensional docking pose of salvianolic acid B with P2Y_12_ receptor.

Computer molecular docking was used to further validate the binding receptor of *dl*‐PHPB, puerarin and salvianolic acid B (Figure 7). The results showed that the bond energies of *dl*‐PHPB and puerarin with P2Y_1_ receptor were − 7.250 and − 6.468 kcal/mol, while the bond energy of salvianolic acid B with P2Y_12_ receptor was −4.039 kcal/mol. Docking results supported the above biological experiment results that *dl*‐PHPB and puerarin specifically acted on the P2Y_1_ receptor and salvianolic acid B specifically acted on the P2Y_12_ receptor.

## 4 Discussion

Some traditional herbal medicines were clinically used for the treatment of cerebral ischemia in China and in most Asia countries. Pueraria lobata and Salvia mitiorrhiza Bunge are often used to treat cardiac and cerebral vascular diseases. Their effective components for anti‐platelets and thrombus are known as puerarin and salvianolic acid A and B, respectively. Puerarin was reported to have similar anticoagulant and antiplatelet effects compared with heparin sodium, and may have a lower hemorrhage risk than heparin sodium [24]. Salvianolic acid B was also reported to dose‐dependently inhibit agonists‐induced platelet aggregation in vitro and in vivo [25]. Recently, *dl*‐PHPB, a derivative that was originally from *Apium gaveolens* Linn, was demonstrated to reduce platelet aggregation potently [9]. Our previous study indicated that the role of *dl*‐PHPB in inhibiting ADP‐induced platelet aggregation is mainly related to the P2Y_1_‐Gq‐PLC pathway from the ex vivo model [11]. However, little information was received about the mechanisms of puerarin and salvianolic acid B inhibiting platelet aggregation.

It has been found that on platelet plasma membrane there are two ADP receptors related to platelet aggregation: P2Y_1_ and P2Y_12_ [26]. Both receptors are G‐protein coupled receptors. There are about 400–1200 2‐MeSADP binding sites on each platelet. About 1/3 of them are P2Y_1_ receptors, and 2/3 are P2Y_12_ receptors [26]. ADP is essential for platelet aggregation due to activation of the P2Y_1_ receptor [27]. Activation of the P2Y_1_ receptor by ADP causes a change in the downstream pathway [28]. The P2Y_12_ receptor is coupled with the Gi protein. When it is activated by ADP, the activity of platelet adenylate cyclase and the level of cAMP are reduced. The platelets from reversible aggregation transform into irreversible aggregation [29].

P2Y_1_ selective receptor antagonists include a series of analogs based on adenine nucleotides, such as MRS2179 [27], which inhibit platelet aggregation and completely antagonize ADP‐induced elevation of intracellular calcium, and does not inhibit AC. MRS2500 is another potent and selective P2Y_1_ receptor antagonist (IC_50_ is 0.95 nM) which can inhibit ADP‐induced platelet aggregation [30]. However, there are still no antiplatelet aggregation drugs targeting P2Y_1_ on the market. P2Y_12_ selective receptor antagonists are very commonly used in clinics. The first generation of the drug is represented by ticlopidine and the second generation of the drug is represented by clopidogrel, which belongs to the thienopyridines and needs to undergo irreversible anti‐platelet aggregation after metabolism in the body [31]. Later, drugs such as ticagrelor entered clinical application, which demonstrated that P2Y_12_ receptor antagonists can inhibit adenylate cyclase, and reduce cAMP levels, thereby inhibiting platelet aggregation [32]. In addition, antipsychotic drugs such as risperidone, clozapine, or haloperidol suppress the aggregation of platelets, Risperidone or clozapine inhibited platelet aggregation via P2Y_1_ and P2Y_12_, whereas haloperidol affected P2Y_1_ only [33]. Tetramethylpyrazine, an alkaloid in Chinese herbal medicine, possesses antiplatelet activity via suppressing P2Y_12_ [34].

In the present study, we investigated in vitro the mechanisms of antiplatelets of *dl*‐PHPB, puerarin and salvianolic acid B using P2Y_1_ and P2Y_12_ transfected HEK293 cells. The advantage of these cells is that they can separately observe the effects of the drugs on P2Y_1_ or P2Y_12_ receptor, and facilitate the determination of specific targets. The HEK293 cells transfected with P2Y_1_ and P2Y_12_ were proved to be successful and reliable by the verification of positive drugs: selective P2Y_1_ receptor antagonist MRS2179 and selective P2Y_12_ receptor antagonist ticlopidine, and therefore can be used to measure the effects of the three compounds on the downstream signal pathways of P2Y_1_ or P2Y_12_.

**FIGURE 8:**
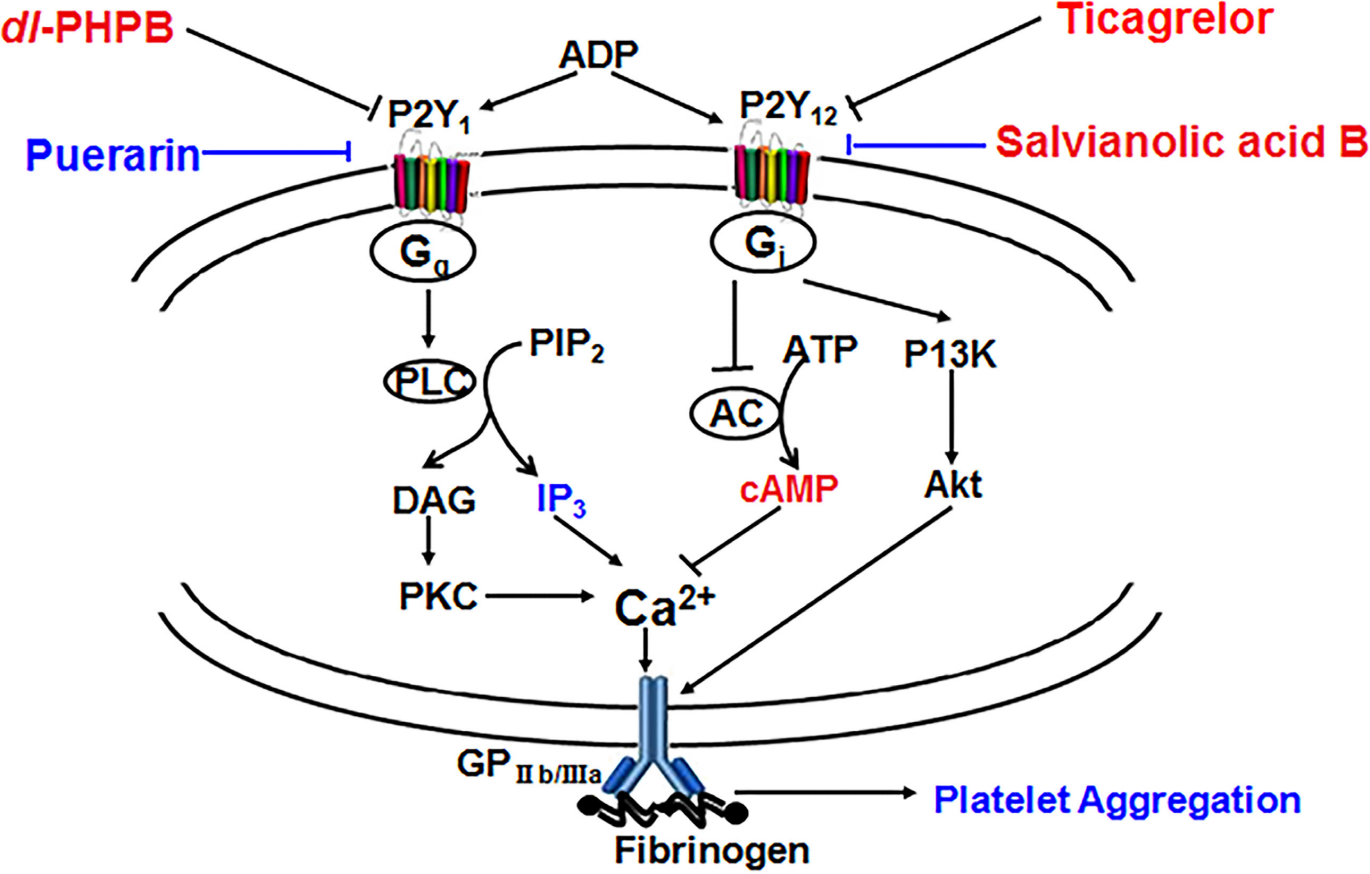
Schematic diagram of the mechanisms of *dl*‐PHPB, puerarin and salvianolic acid B against platelet aggregation induced by 2‐MeSADP. *Dl*‐PHPB and puerarin inhibit the Gq‐PLC‐IP_3_‐Ca^2+^ signal transduction pathway, reduce intracellular Ca^2+^ and decrease platelet aggregation. However, salvianolic acid B as well as ticagrelor inhibit the Gi‐AC‐cAMP signal transduction pathway and enhance cAMP and decreases platelet aggregation. This figure was modulated on the basis of our former published study [11].

IP_3_, cAMP and Ca^2+^ are important second messengers downstream of the P2Y_1_ and/or P2Y_12_ receptors [35, 36, 37]. Both *dl*‐PHPB and puerarin reversed the increased IP_3_ induced by 2‐MeSADP, but salvianolic acid B had no effects on IP_3_ increase. Considering IP_3_ is the major signal factor downstream of the P2Y_1_ receptor and increased IP_3_ could enhance intracellular [Ca^2+^]_i_ and leaded to platelet aggregation. These results indicated that salvianolic acid B definitely did not target at P2Y_1_ receptor and *dl*‐PHPB and puerarin could act on P2Y_1_ receptors. The cAMP measurement further confirmed the above speculation. Both *dl*‐PHPB and puerarin did not affect the decreased cAMP induced by 2‐MeSADP while salvianolic acid B could obviously enhance the cAMP content. These results further demonstrated that the targets of *dl*‐PHPB and puerarin are not related to the P2Y_12_ receptor signal pathway. Conversely, salvianolic acid B might be a P2Y_12_ receptor blocker which affected platelet aggregation by blocking the binding of P2Y_12_ receptor to ADP, these results were consistent with previous reports [38]. In addition, different from salvianolic acid B, anti‐platelet aggregation of *dl*‐PHPB and puerarin is associated with inhibition of intracellular [Ca^2+^]_i_. Based on the above results, *dl*‐PHPB and puerarin can reduce the IP_3_ content and the cytosolic intracellular [Ca^2+^]_i_ of cells, and inhibit platelets activation by antagonizing P2Y_1_ receptor, which in turn inhibits 2‐MeSADP‐induced platelet aggregation. Their target is different from the current antiplatelet drug clopidogrel or ticagrelor. Salvianolic acid B inhibits 2‐MeSADP‐induced platelet aggregation by affecting P2Y_12_ receptor and increasing cAMP content. Its target is the same as clopidogrel or ticagrelor. Schematic diagram of the mechanisms of *dl*‐PHPB, puerarin and salvianolic acid B against platelet aggregation induced by 2‐MeSADP can be seen in Figure 8.

In clinical practice, it is often recommended to use aspirin or clopidogrel [39, 40] to prevent platelet aggregation and thrombus formation. However, both have some serious adverse reactions and resistance [41], especially, they might induce the risk of bleeding in the treatment of patients. However, *dl*‐NBP, the parent drug of *dl*‐PHPB, has been used in clinics for more than 15 years, and puerarin and salvianolic acid B have even been used for several decades with clear anti‐platelet effects, respectively. Almost no side effects of bleeding were seen, indicating the good safety of above natural products. It is still necessary to explore an oral antiplatelet agent targeting P2Y_1_ and/or P2Y_12_ for the treatment of thrombotic diseases to overcome the drawbacks of these clinical drugs and achieve a fast onset of action with less bleeding risk [42].

Our present study clarified the binding targets of these natural products to prevent thrombus formation. Our study also showed an opportunity and a new target for drug development, because as far as we know, there is no listed drug for anti‐platelet targeted P2Y_1_ receptors up to now. Actually, the dual‐target antagonist of P2Y1 and P2Y12 was considered and developed as an oral antiplatelet agent [42]. In addition, it is helpful for promoting relevant research and developing new anti‐platelet drugs from traditional Chinese herbal medicine.

In conclusion, *dl*‐PHPB, puerarin and salvianolic acid B can inhibit ADP‐induced platelet aggregation in vitro and in vivo. *Dl*‐PHPB and puerarin inhibit the Gq‐PLC‐IP_3_‐Ca^2+^ signal transduction pathway, reduce IP_3_ and intracellular [Ca^2+^]_i_ and decrease platelet aggregation via acting on the P2Y_1_ receptor. However, salvianolic acid B, like ticagrelor, inhibits the Gi‐AC‐cAMP signal transduction pathway, enhances cAMP and decreases platelet aggregation by acting on P2Y_12_. The P2Y1 receptor alone or in combination with the P2Y12 receptor is a new direction for the development of antiplatelet aggregation drugs in the future.

## Author Contributions

Xiaoliang Wang and Yiying Li designed the experiments; Yiying Li, Weiping Wang, Jie Cai and Nan Feng performed the research; Yiying Li, Weiping Wang, Shaofeng Xu and Ling Wang analyzed the data; Yiying Li, Weiping Wang and Xiaoliang Wang wrote the paper.

## Conflicts of Interest

The authors declare no conflicts of interest.

## Data Availability

The data that support the findings of this study are available from the corresponding author upon reasonable request.
